# Micronutrient Powders for Infants and Young Children

**DOI:** 10.9745/GHSP-D-21-00263

**Published:** 2021-06-30

**Authors:** Stephen Hodgins, Rolf Klemm

**Affiliations:** a Editor-in-Chief, Global Health: Science and Practice Journal, and Associate Professor, School of Public Health, University of Alberta, Edmonton, Alberta, Canada.; b Helen Keller International, New York, NY, USA.

## Abstract

Providing standalone micronutrient products for household use is not an easy strategy, but under the right conditions, it can work. To be effective, micronutrient powder programs require robust commodity logistics and support of uptake and adherence.

See related article by Dusingizimana et al.

## STRATEGIES TO DELIVER MICRONUTRIENTS TO AT-RISK POPULATIONS

Micronutrient deficiencies, notably of vitamin A, iron, and zinc, have occupied a prominent place on the child health and nutrition agenda over the past 3 decades, and for good reason: there has been robust evidence on the population burden of these deficiencies, particularly in low- and lower-middle-income countries, as well as on their contribution to morbidity, mortality, and compromised developmental outcomes. The situation has been improving, but there is still evidence of widespread deficiencies of these and other micronutrients, especially in sub-Saharan Africa and South Asia.^1^ Furthermore, intervention trials have demonstrated unequivocal reductions in risk of under-5 mortality for vitamin A supplementation^2^ and reductions in respiratory infection and diarrhea incidence and all-cause mortality for zinc supplementation.^3^

So, we have a significant problem, and we also have specific *technical interventions* for which there is evidence for efficacy. That's a good thing…but it doesn't necessarily mean we have readily available real-world *delivery strategies* that can fix these problems quickly at scale.

The child health and nutrition community has consistently upheld dietary diversity as the ideal. With rising living standards, there have been concomitant improvements not only in protein-energy adequacy but also in micronutrient intake. Nevertheless, the Food and Agriculture Organization has documented that the cost of a nutrient-adequate diet exceeds the international poverty line,^4^ with the result that about 3 billion people still cannot afford the minimum cost of a healthy diet. So, appropriately, the child health and nutrition community has sought to accelerate improvements, beyond what improved living standards alone can contribute. This has been achieved, in part, through the development of strategies aiming to deliver key micronutrients in ways that effectively reach whole populations and especially segments of the population most at risk for severe deficiencies and their consequences.

## FOOD FORTIFICATION AND SUPPLEMENTATION

One of the most momentous public health achievements of the 20^th^ century has been the near-elimination of iodine-deficiency-related compromise in cognitive development (with cretinism as its extreme manifestation), achieved largely through salt iodization. Incorporating micronutrients into widely consumed, commercially processed food products—*food fortification*—has proven a highly effective strategy, provided that certain key conditions are met, notably that suitable food products are available as fortification vehicles, and that cost, taste, and appearance are not affected.

In the trials that first demonstrated the contribution of these micronutrients to child morbidity, mortality, and compromised development, the delivery strategy used was *supplementation*. Compared with commercial food fortification, supplementation is inherently a more difficult way to achieve improvements in population health and nutrition outcomes, as it requires (1) logistical arrangements to ensure a reliable supply of a micronutrient commodity to end-users, and (2) a level of user adherence sufficient to produce a health or nutrition benefit. Furthermore, these conditions need to be sustained continuously until the underlying nutrition status of the population is adequate, through dietary intake.

Compared with commercial food fortification, supplementation is inherently a more difficult way to achieve improvements in population health and nutrition outcomes.

When certain requirements are met, it has proven feasible to meet both of these conditions under real-world programs at scale. Notably, the use of iron-folate supplements by pregnant women has been achieved at relatively high coverage, in some countries, although in most, coverage remains low.^5^ In this instance, provision of the commodity to the end-user has been done, taking advantage of an available, generally high-coverage contact with the primary health care system—antenatal visits (ANC). Among 47 countries in sub-Saharan Africa and South and South East Asia for which ANC visit data are available from Demographic and Health Surveys (DHS) conducted over the past 10 years, in 38 of them, more than 85% of women who had given birth over the previous 5 years reported having made at least 1 ANC visit.^6^ Such contacts provide an opportunity for health workers to dispense the product and to counsel their pregnant clients on the rationale for its use and how to minimize side effects. Pregnant women, themselves, are generally highly motivated to adopt practices they believe will help protect and strengthen their unborn babies, and, in this instance, they are asked to adhere to daily supplement use only for a few months. So, the provision of standalone micronutrient products for household use is not an easy strategy, but under the right circumstances, it can work.

Another relatively successful use of such a strategy has been periodic distribution of high-dose vitamin A to infants and children aged 6–59 months, in countries where this deficiency remains common and regular service delivery is challenging. Using a twice-annual, campaign-style delivery strategy (sometimes piggy-backed on Supplemental Immunization Activities), many countries have been able to reach the majority of children in this age group. But this is a demanding and costly strategy that may interfere with routine service delivery.^7^ In recent years, some key stakeholders have shown declining support,^8^ and, indeed, for some populations it may now be appropriate to review whether this effort should be maintained.^9^^,^^10^

## WHAT ABOUT MICRONUTRIENT POWDERS AS A STRATEGY FOR ADDRESSING MICRONUTRIENT DEFICIENCIES IN INFANTS AND YOUNG CHILDREN?

Micronutrient powders (MNPs) have been promoted as a home-based strategy, controlled by the caregiver, to improve dietary quality for infants and young children. In settings where the use of MNPs has been promoted (framed as home or point-of-use “fortification”), clearly, there has been a significant problem of deficiencies of the key micronutrients included in these powders. And, in principle, if these products are reliably consumed several times a week, we would expect benefits (largely, improved iron status) in line with what has been documented in the published trials.^11^ It has been demonstrated that where there are committed, adequately funded, and well-managed implementers along the whole supply chain down to the community level, to support MNP logistics, and where context-specific challenges with adherence are adequately addressed, it is possible to achieve relatively high effective coverage^12^ (i.e., a large proportion of those who could, in principle, benefit from such an intervention actually do.)^13^ But that is a challenging set of conditions to be met for the intervention to produce its desired population health and nutrition goal.

The article by Dusingizimana et al.^14^ in this issue of *Global Health: Science and Practice* documents the performance of an MNP program in Rwanda. As they report, MNPs have been implemented in Rwanda with support from United Nations Children's Fund (UNICEF) and other partners. First adopted as policy, the program was fully scaled up across the country by 2017. Under the program, sachets of MNP are delivered to the district by UNICEF and the Ministry of Health; they are then distributed to health centers and then to community health volunteers, who have the responsibility to dispense them to households with infants and children aged 6–23 months and counsel mothers on their use.

In this study,^14^ program performance was assessed in Rutsiro district, 1 of 19 in the first wave of scale-up. The district was selected for this study primarily because it was found to have a particularly poor baseline nutrition status. The district was typical regarding the degree of outside support received for MNP implementation.

The authors found evidence of relatively good program reach: almost two-thirds of mothers (64%) reported ever having used MNPs. But effective coverage was much lower: 38% reported having used MNPs at least once over the previous week, and use was considerably lower among those aged 6–11 months than among those aged 12–23 months. Furthermore, use was markedly lower among households with high “hunger scores”—those in which MNP use would be likely to produce the greatest benefit. Digging into the causes of the disappointing findings, predictably the authors found problems with commodity logistics and adherence. Supplies were not reliably available, and many mothers either did not see the value in MNP use or found they affected palatability of the thin porridges commonly given to infants aged 6–11 months in this setting.

Rwanda is widely recognized as an exemplar for the performance of its primary health care programs and, arguably, has been the biggest MNP success story in sub-Saharan Africa. It remains the one country on the continent to have fully scaled up this intervention. It is less clear, however, what impact this has made. Among young children aged 12–23 months, comparing findings from the 2010 Rwanda DHS^15^ to those of the 2019–2020 DHS^16^ (i.e., from before the introduction of MNPs to after full national scale-up was achieved), there was a modest decline in mild anemia (HgB 10–10.9), from 31% to 26% but no change in moderate to severe anemia (20% in both surveys). Note that with high-fidelity delivery and adherence, efficacy trials have shown a one-third reduction in anemia.^11^

There were other notable improvements in child nutrition status over this interval, not attributable to MNPs: of children aged 12–23 months, the proportion stunted (<–2SD height for age) dropped from 49% to 36%. So, even the modest decline in mild anemia cannot necessarily be attributed to the MNP program alone.

It needs to be acknowledged: MNP programs are a comparatively heavy lift, requiring—for their effectiveness—robust commodity logistics and behavior change efforts supporting uptake and adherence. Neither of these conditions can be easily achieved and maintained at scale. This is certainly not unique to MNP programs; other efficacious nutrition and health interventions also have onerous supply- and demand-side requirements that undermine the feasibility of achieving sustainable high-coverage delivery at scale.

MNP programs are a comparatively heavy lift, requiring—for their effectiveness—robust commodity logistics and behavior change efforts supporting uptake and adherence.

## ROUTINE DELIVERY AT SCALE: THE DEVIL'S IN THE DETAIL

With management oversight vigilance and dedicated resources, many delivery strategies can give promising results when implemented at relatively small scale. But such results are often poor predictors of program performance at scale, under routine, institutionalized conditions. MNP program efforts have been successful, in the sense that such programs have been introduced in many countries over the past decade. However, it is much less clear what they have contributed to improving mortality, morbidity, and developmental outcomes—at scale. As noted by Pelletier et al.,^17^ even with a decade of efforts to introduce and scale up this intervention, most of the programs documented in the peer-reviewed literature have been of modest scale, implemented over relatively short periods, and dependent on significant external support. Review of large-scale program experience^18^ has found little evidence on how to effectively reach a large proportion of those targeted and achieve high adherence, when implementing under routine, at-scale conditions.

Evidence from studies such as that reported on by Dusingizimana et al. suggests that, in many instances, these programs are not achieving the population health impacts hoped for by their promoters. In settings where it will not be feasible to ensure reliable commodity supply or adequate adherence support, population-level impacts will not be attained.

## TIME TO RETHINK?

In the presence of a clear need—in this case, a high burden of serious morbidity, mortality, and compromised development outcomes—and having in hand an intervention proven to be efficacious in addressing that need, it is certainly warranted to make serious efforts to develop and test practical delivery strategies that may have the potential for high coverage under real-world conditions. Such development and testing need to be done on an iterative basis, beginning with smaller-scale proof-of-concept piloting, progressing to tests of delivery effectiveness at a progressively larger scale and more routine conditions. And, as difficult as it may be for those championing a potentially high-impact intervention, we also need to be willing to step back and look critically at our efforts and, when necessary, go back to the drawing board.

Rwanda is a notable high performer with regard to the effective delivery of primary health care interventions. But, even in Rwanda, it is legitimate to ask if the effort expended to date, to introduce, scale up, and sustain MNPs has been worth it, given the modest population impact that can be attributed to this effort.

*Has the juice been worth the squeeze?*

## Notes

**Peer Reviewed**

**First published online:** May 27, 2021.**Cite this article as:** Hodgins S, Klemm R. Micronutrient powders for infants and young children. *Glob Health Sci Pract*. 2021;9(2):216-219. https://doi.org/10.9745/GHSP-D-21-00263
